# Optimizing Stimulation and Analysis Protocols for Neonatal fMRI

**DOI:** 10.1371/journal.pone.0120202

**Published:** 2015-08-12

**Authors:** Rhodri Cusack, Conor Wild, Annika C. Linke, Tomoki Arichi, David S. C. Lee, Victor K. Han

**Affiliations:** 1 Brain and Mind Institute, Western University, London, Canada; 2 Children’s Health Research Institute, London, Canada; 3 Department of Perinatal Imaging and Health, King’s College London, London, United Kingdom; 4 Department of Bioengineering, Imperial College London, London, United Kingdom; Universiteit Gent, BELGIUM

## Abstract

The development of brain function in young infants is poorly understood. The core challenge is that infants have a limited behavioral repertoire through which brain function can be expressed. Neuroimaging with fMRI has great potential as a way of characterizing typical development, and detecting abnormal development early. But, a number of methodological challenges must first be tackled to improve the robustness and sensitivity of neonatal fMRI. A critical one of these, addressed here, is that the hemodynamic response function (HRF) in pre-term and term neonates differs from that in adults, which has a number of implications for fMRI. We created a realistic model of noise in fMRI data, using resting-state fMRI data from infants and adults, and then conducted simulations to assess the effect of HRF of the power of different stimulation protocols and analysis assumptions (HRF modeling). We found that neonatal fMRI is most powerful if block-durations are kept at the lower range of those typically used in adults (full on/off cycle duration 25-30s). Furthermore, we show that it is important to use the age-appropriate HRF during analysis, as mismatches can lead to reduced power or even inverted signal. Where the appropriate HRF is not known (for example due to potential developmental delay), a flexible basis set performs well, and allows accurate *post-hoc* estimation of the HRF.

## Introduction

Perinatal brain injury can be caused by adverse events such as hypoxia, hemorrhage and stroke [1], and brain development can be altered by extremely premature birth [2]. The consequences vary from infant to infant: in some cases function develops normally, even though substantial brain injury is seen with ultrasound or anatomical MRI; in some, it becomes apparent in the first postnatal years that basic functions (i.e., motor, audition or vision) are affected; and in others higher-level cognitive or behavioral impairments emerge at preschool age or later. Unfortunately, it is difficult to assess brain function [3], and the current attitude is often to “wait and see” what problems emerge. This makes it difficult to focus care and support on infants that need it most, prevents targeted interventions early (when plasticity is high and they are most effective), and impedes the development of new interventions.

**Fig 1:**
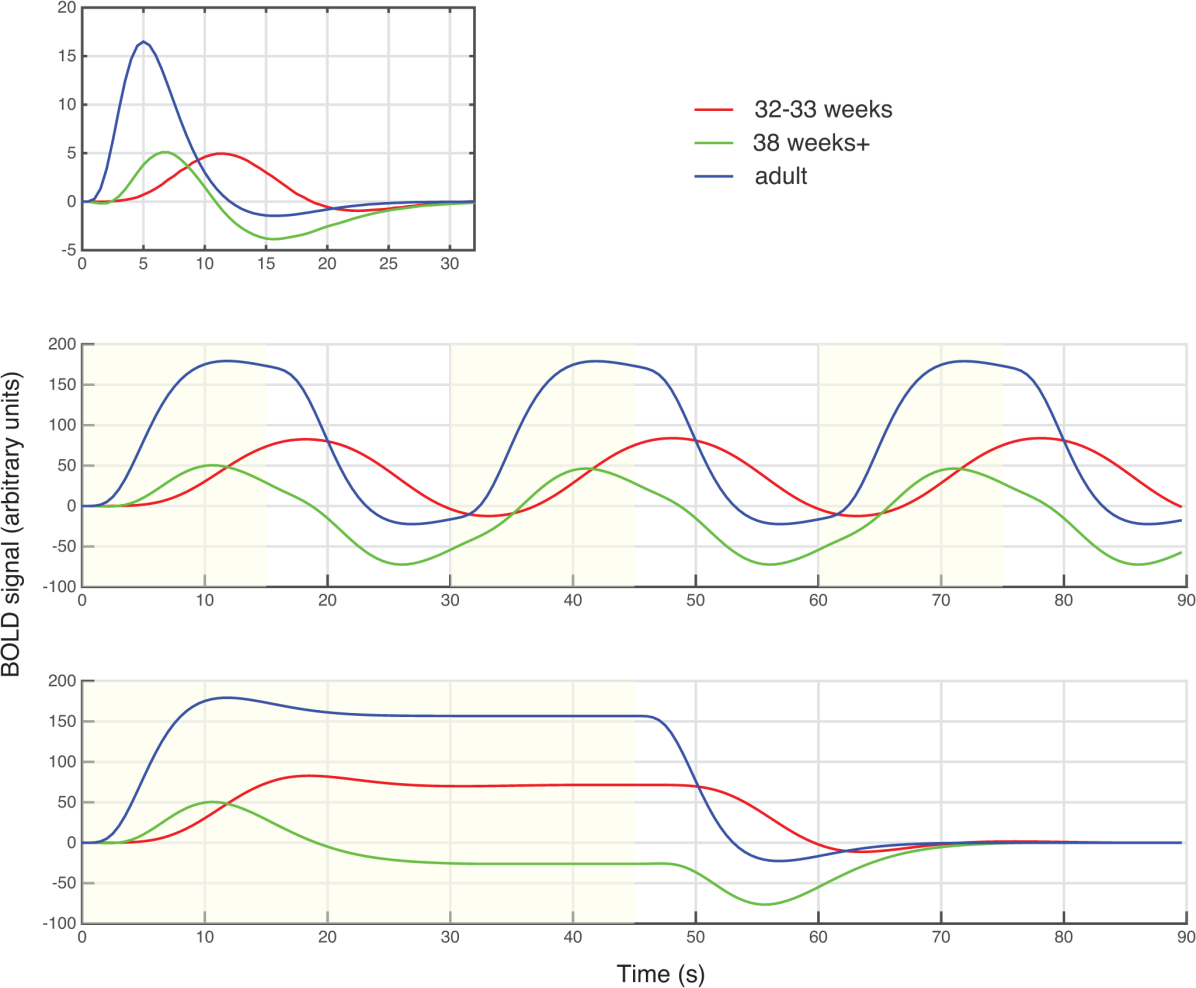
(a) The hemodynamic response (HRF) to a brief 1-second stimulation at three ages (Arichi et al, 2012). In adults the HRF is dominated by a positive peak, while at 38 weeks gestational age (GA) neonates have positive and negative peaks of similar magnitude. At 32 weeks GA the HRF is dominated by a positive peak, but it is much delayed. (b) The form of the HRF affects the power of different stimulation designs. To illustrate this, the response to a 30s-long cycle of stimulation (yellow) and rest was calculated by convolving the HRFs with a boxcar. For this design, at all three ages, there was substantial modulation of the BOLD signal through time. The signal in adults and 38 week infants was highly correlated, but at 32 weeks the signal has a different phase. (c) In contrast, for 45s of stimulation is followed by 45s of rest, the 38 week infants only have small peaks of modulation in the BOLD signal, and so much reduced power would be expected.

A fundamental challenge in assessing function is the limited behavioral repertoire of young infants. A promising solution is to use neuroimaging to assess brain function, as this doesn’t require a behavioral response. fMRI has been used in neonates to measure the brain activity evoked by visual [4,5], auditory [6–8] and somatosensory [9] stimulation. However, there are a number of methodological challenges that need to be addressed to improve the robustness of the signal [10]. This manuscript focuses on one key challenge. fMRI does not measure neural activity directly, but instead a hemodynamic signal sensitive to blood oxygenation. The hemodynamic response is delayed relative to the neural activation, likely due to both the time taken for neurotransmitters to signal opening of arterioles, and for blood to flow through the capillary bed and into draining veins [11]. The temporal profile of this delay is known as the hemodynamic response function (HRF). In adults, if a stimulus evokes a short burst of neural activity there is a peak in the HRF around 5 seconds later [12]. In infants the HRF has a different size and shape (Fig 1A), although there has not yet emerged a consensus across studies using fMRI [13,14,15], near-infrared spectroscopy (NIRS) [16, 17, 18], and optical imaging [19]. At term age or earlier, the HRF has been shown to peak later (6-12s) in both mice [14,19] and humans [13]. The polarity of the HRF has sometimes been found to be reversed [6,19] and is sometimes biphasic, with positive and then negative lobes [13,19].

We consider two consequences of the altered HRF. First, conventional fMRI analyses proceed by generating a model of the expected signal, and comparing this to the fMRI time-course. If an incorrect model of the HRF is used, power to detect brain activity may be reduced. A second important consequence is more subtle: the altered HRF will affect which protocols of stimulation will yield the best sensitivity. As an illustration, Fig 1B and 1c show the expected HRF for two different block designs that yield quite different power. The HRF blurs the response to events close in time–in other words, it acts as a low-pass (temporal smoothing) filter, so reducing the power of faster designs. However, opposing this, the noise in fMRI drifts over time, and contains greater power at lower frequencies. The effect of this is that if the fMRI stimulation changes on a slower cycle it will be harder to detect the signal against the larger amplitude of noise. These two effects are such that each dominates at extremes, and in between there is an optimum. Measurements in adults have suggested that the maximal power is obtained with on/off stimulation cycles of around 24–60 s in duration [20,21,22, http://www.mccauslandcenter.sc.edu/CRNL/tools/fmrisim). However, no such optimization has been done for infants, and this forms the second goal of our paper.

## Methods

### Overview

The power of various stimulation protocols and analysis methods was assessed using simulation: standard statistical measures (t- and F-statistics) were computed to indicate how well a synthesized BOLD fMRI signal could be detected in well-characterized fMRI noise, given a particular model of the hemodynamic response. A realistic model of fMRI noise was obtained from analysis of adult and neonate resting-state fMRI data. Simulated signal was obtained by convolving a range of stimulation paradigms with three HRFs previously measured empirically in adults and infants (Arichi et al., 2012). Analyses then proceeded assuming either the correct or mismatching HRFs.

### Statistics of noise

#### Characterizing Noise: Resting-state MRI acquisitions

The power in an fMRI analysis (and thus the accuracy of any simulation) depends not just on the strength of the signal but on the nature of the noise. For a model of noise in the brain, we used fMRI data acquired during resting in adults (N = 26) and newborns (N = 5). The adult data were acquired using a 3T Siemens Tim Trio MRI scanner and the infant data using a 3T Philips Acheiva MRI scanner.

The adult data was the Marguilies subset of the fc1000 public domain resting-state dataset (http://www.nitrc.org/frs?group_id=296), obtained in 13 males and 13 females, comprising 195 gradient-echo EPI scans with TR = 2.3 s, 34 slices, a matrix size of 70x70, voxel size 3x3x4mm. An MPRAGE T1-weighted anatomical image was acquired in each adult, matrix size 176x256x256, 1-mm isotropic voxels.

5 neonates (3 female, 2 male; post-menstrual age at scan 40.3 +/- 2.4 weeks) were scanned at the Hammersmith Hospital. They were all born prematurely and had no overt brain injury (Table 1). One infant was diagnosed with chronic lung disease. fMRI comprised 256 gradient-echo EPI scans with TR = 2 s, 22 slices, a matrix size of 80x80, voxel size 2.5x2.5x4mm. Due to developmental differences in tissue composition and water content, the relaxation times of grey and white matter markedly differ in neonates in comparison to standard adult values [23], and T1-weighting gives poor contrast, so a T2-weighted sequence was used for anatomy (matrix size 256 x 256, 95 slices, 0.86x0.86x1mm).

**Table 1:** Demographic and clinical details of the neonates.

Subject	Gestation at birth	Post-Menstrual age at scan	Birth weight	Head circumference at birth	Intra-ventricular haemorrhage(Levene classification)*	Days on ventilation	Chronic Lung disease	sepsis
**1056**	41+1	29+1	970	25.8	none	1 day	no	no
**1070**	43+3	25+5	815	23	1	6 days	yes	no
**1131**	41+4	39+0	3571	34.5	none	none	no	no
**922**	40+2	33+5	2260	33	none	none	no	no
**946**	36+0	36+4	2176	32	none	none	no	no

*Arch Dis Child 1982;57:*410–417 10*.*1136/adc*.*57*.*6*.*410*.

#### Resting-state analysis

Both infant and adult data were processed using the parallel-processing *automatic analysis* software [24]) to pipeline SPM 8 and custom-matlab modules. Processing comprised motion correction and smoothing with a Gaussian kernel of FWHM 10mm. Note that we did not regress out signals from the white matter or ventricles (common in resting-state analyses) as this is not typically done in fMRI activation studies, which we wished to simulate.

The time-series statistics were characterised in grey matter, as this is of most interest in activation studies. For the adults we normalized and segmented anatomical images and created a mask comprising voxels with a greater than 50% probability of grey matter; while in the infants, due to the difficulty of normalization and segmentation, we manually drew a grey-matter mask on a portion of the right lateral frontal lobe of the un-normalized anatomical images. As can be seen in the results, when motion was of a similar magnitude, similar noise statistics were obtained in infants and adults.

#### Summarizing the statistics of the noise

It is well established that fMRI noise is strongly auto-correlated (i.e., the noise on one scan predicts the next), and has strong power at low frequencies [25]. It is often characterized as having a form

$$p(f)=\frac{A}{f^{x}}+W$$

*where p = power, f = frequency, x, A and W are constants*

The exponent *x* has been taken to be 0.67 [26] and 1 [12,25,27]. We calculated the power spectrum for each grey matter voxel, and then averaged this across voxels.

#### Synthesizing noise with fMRI statistics using a simple model

To ensure that the noise was well characterized, and to allow for simple extension and replication, we wished to synthesize noise with the same statistics as the measured resting-state noise. Surprisingly, we did not find an existing tool for this, and further, there was no consensus on the best approach. Thus, in pilot work using the adult data, we tested three approaches to synthesizing noise with fMRI-like statistics. The first was to measure the lag-1 autocorrelation of the fMRI time-series, and then to synthesize noise with this autocorrelation. This time domain model was found to be wanting, as it underestimated the magnitude of very low frequency fluctuations in the power spectrum, which have an important impact on the power of slow block designs. The second method was to measure the power spectrum of the fMRI noise, and to synthesize noise with this power spectrum. The measured adult noise was fit well by a linear model of *log (power)* against *log (frequency)*. The frequency domain synthesis comprised magnitudes taken from this model of the power spectrum and random phases, which was then transformed to the time domain using a Fourier Transform. The resulting noise had similar amplitude histograms, and appeared visually similar, to the original noise. The third method formed a balance between the time-domain and frequency domain methods above, and was based on the discrete wavelet transform (DWT) analysis of fMRI noise by [28]. We performed a multi-scale DWT using the Daubechies wavelet, either of order 1, 2 or 4, with 7 levels of decomposition. We then summarized the distributions of the wavelet components at each of these levels. To synthesize noise, random wavelet domain components were generated from these distributions and the wavelets reconstructed into the time domain. This was found to give similar final results to the synthesis of fMRI noise in the frequency domain above, but requires a model of the noise with many more parameters. We thus report only the simpler yet equally effective frequency-domain noise synthesis.

### Power as a function of paradigm block length and HRF

#### Choice of hemodynamic responses

Arichi et al (2012) measured the HRF in pre-term and term infants, and adults, for a 1-second somatosensory stimulation. The resulting curves show a broad range of morphology–delayed in pre-term infants relative to adults, but still broadly monophasic; and biphasic in term infants (Fig 1a). For our simulations, we used Arichi’s hemodynamic measurements for adults, premature infants of 32 weeks gestational age (GA), and term infants at 38 weeks GA. These are the only measurements of the hemodynamic response to a brief event in infants (that we know of), and they have a broad range of morphology, ensuring our simulations span a range of possibilities.

#### Simulated stimulation

Block designs, in which for example a stimulation block is alternated with a rest block, are simple and robust [29]. We analyzed the power of block designs spanning 24 block lengths, from rapid (4-s cycle period comprising 2 s on, 2 s off) to slow (97 s cycles comprising 48.5 s on, 48.5 s off), on a log scale (i.e., 2^2 to 2^6.6). To simulate the fMRI signal that would be obtained in adults, and infants of 32 and 38 weeks GA, box-cars corresponding to each block design were convolved with the corresponding empirical HRF. For each paradigm/HRF combination, this signal was combined with 1000 samples of synthesised noise.

#### Matching and mismatched HRFs

In fMRI studies, analyses typically begin with the creation of a model of the signal, by convolving the expected neural activity with the HRF. In the literature, many authors have used mismatched responses, for example by assuming the HRF of infants is the same as adults. To assess the effect of a mismatch, we analyzed the data simulated with each of the three HRFs using three different models (each of the possible HRFs). The fit of each model to the simulated data was estimated using the general linear model using expectation-maximization in SPM 8. Each model contained two columns: the convolved boxcar, and the session mean. The power of the model to detect the simulated activation was assessed using an SPM-T statistic for the first of these two columns.

### Flexible basis set

In practice, it may often be unclear which HRF is appropriate for a participant. Infants develop at different rates, especially when they have faced some challenge such as being born with very low birth weight, or requiring an extended period of mechanical ventilation. It is not yet known under what circumstances “HRF age” mismatches from “chronological age”, or the extent of this mismatch. We thus developed a flexible basis set, using a procedure from FSL’s “FLOBS” (http://fsl.fmrib.ox.ac.uk/fsl/fslwiki/FLOBS). First, the three HRFs were fitted to a set of half-cosine functions, allowing each to be parameterized by a set of delays and amplitudes. Second, in this parameter space we interpolated between the 32- and 38-week HRFs, and the 38 week and adult HRFs. Third, we reconstructed a set of HRFs, and conducted Principal Component Analysis. The top three components accounted for >99% of the variance, and fit well the original Arichi or interpolated HRF functions. They thus form an effective flexible basis set. A model using this basis set contained four columns: the boxcar convolved with each of the three basis functions, and the session mean. The power of the model to detect the simulated activation was assessed using an SPM-F statistic for the F-contrast constructed as the identify matrix. To facilitate comparison to the T statistic, this was then transformed by: adjusting for the difference in the degrees of freedom in the two models, and taking the square root. The resulting value thus corresponds to the T value from the two-column model that has the same significance as the F statistic in the four-column model.

Power curves were constructed by calculating the appropriate t- or F-statistic for each of three sets of simulated data, modeled with four possible basis sets, for each possible paradigm length (for a total of 12 curves). Cubic interpolation with an accuracy of 0.1s was used to find the peak of these curves, and hence the optimal paradigm period for each combination of actual and predicted HRF.

To assess the quality of the HRFs as estimated from the flexible basis set, we reconstructed the HRF by weighting the three basis functions with their estimated beta values, and calculated the correlation over the duration of the true HRF (32.5 s). These correlation values were then Fisher transformed to make them Gaussian, the mean and standard deviation calculated, and the upper and lower bounds were then back transformed for display.

## Results

### Statistics of noise

**Fig 2:**
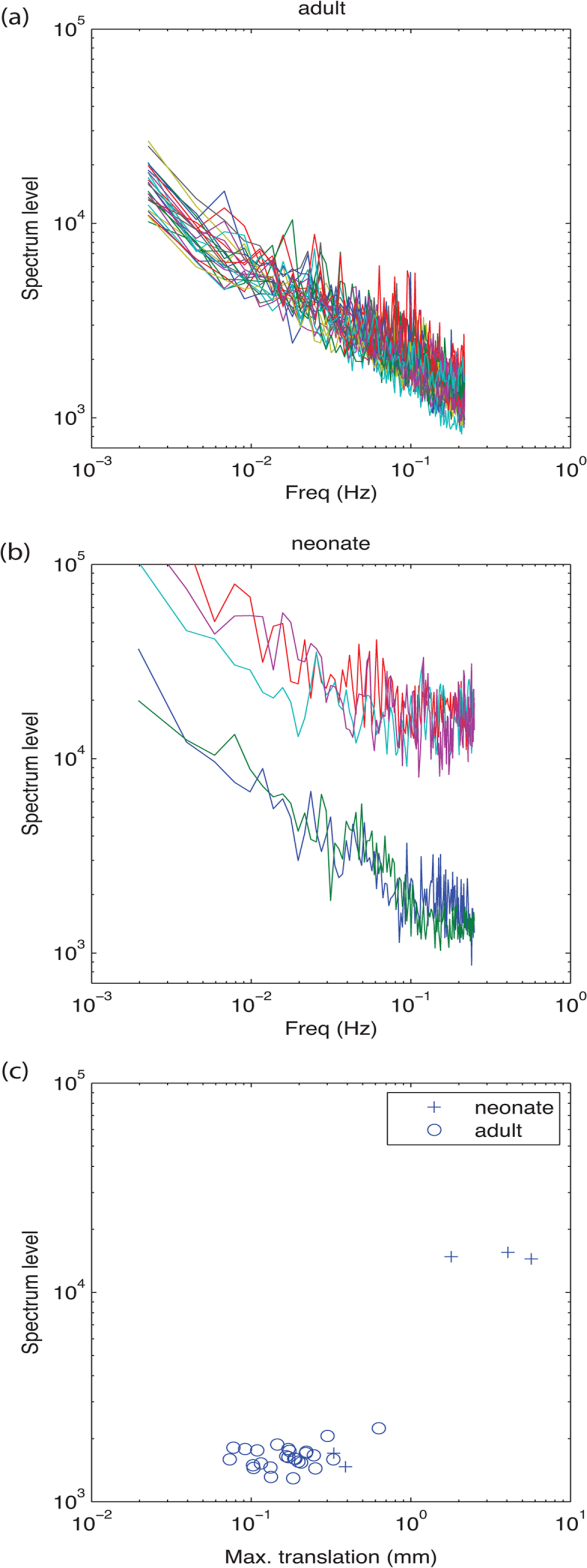
To formally assess the efficacy of different designs, it is necessary to establish the characteristics of the noise in the fMRI signal. In both adult (a) and neonatal (b) participants, spectra were well fitted by flat-frequency spectrum component combined with a 1/f component. In 2/5 neonates the overall level of noise was similar to adults, but in 3/5 neonates, the noise level was elevated. This increased level of noise was associated with larger movement in these neonates (c).

The power spectrums of the noise derived from resting state data for adults and neonates are shown in Fig 2. The adult noise was well described by a flat-spectrum (white) component, added to a 1/f component. The neonate noise was similar in form, and for 2/5 participants, similar in amplitude to adults, despite the different scanners and protocols. However, for 3/5 it had substantially higher amplitude. To investigate whether this was related to greater movement in these infants, we plot in panel (c) a summary measure of the maximum movement, against the noise level averaged across frequency. It can be seen that the 3/5 infants with greater noise did move more than the adults, but when movement was similar to adults, so the noise magnitude was similar.

The noise average fit across adults was $P(f)=\frac{0.1636}{f}+4.86$ where f is the frequency in Hz, and P the amplitude spectrum level. This was the model used to resynthesize fMRI noise for both adults and infants. We confirmed that the resulting noise had a similar distribution of standard deviations to the fMRI noise.

### Power as a function of paradigm block length and HRF

**Fig 3:**
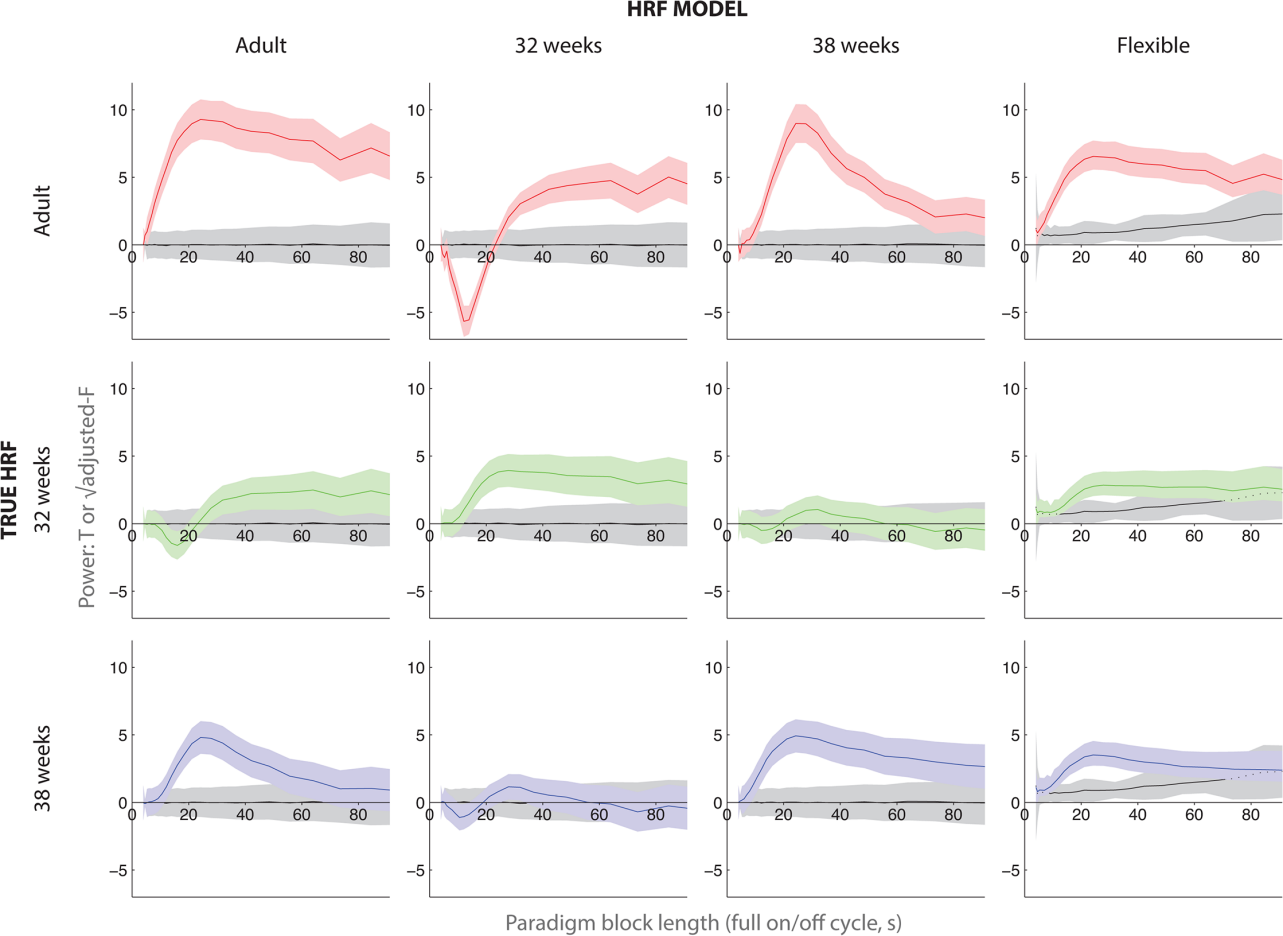
Simulation was used to assess the power of block designs varying in stimulation/rest cycle duration (x-axis of each subplot). Higher values (y-axis of each subplot) correspond to greater statistical power. For example, in the top left subplot, peak statistical power was obtained with a block design of total cycle length 24 s (i.e., 12 s stimulation, then 12 rest). These calculations were repeated for matched or mismatched HRFs during analysis (three rows–true HRF used in simulation; four columns–HRF used for modeling). For the first three HRF columns, the SPM-T statistic is reported. For the flexible HRF model in the fourth column, the square root of an adjusted F statistic is displayed so that the corresponding p-value will match that of the T statistics. The mean +/- one standard deviation is shown. The grey bars show the distribution of fits to null data.

Fig 3 shows the resulting power of the paradigms as a function of block length (full on-off cycle), for three “true” signal HRFs and four HRF models. The model predicted greatest power in adults at a paradigm period of 24s. A slightly longer paradigm period (27.9s) was optimal with the 32 week HRF signal and model, and one similar to the adults at 38 weeks (24.3s). Paradigm power fell off with protocol period more steeply at 38 weeks (when the HRF is biphasic) than in adults or at 32 weeks.

Not surprisingly, greatest power was obtained across all paradigm periods and for all true HRFs when the correct model HRF was used. Modeling with a mismatched HRF had a strong effect on power, in two cases (32 and 38 weeks GA mismatched with each other in either direction) almost entirely eliminating sensitivity to the signal. At 32 weeks GA, power shows a negative trend for rapid protocol alternations (around 16s) when the analysis was conducted using the adult HRF. The relationship of this to reports in the literature of “negative activations” in neonatal fMRI studies is addressed in the Discussion.

### Flexible basis set

**Fig 4:**
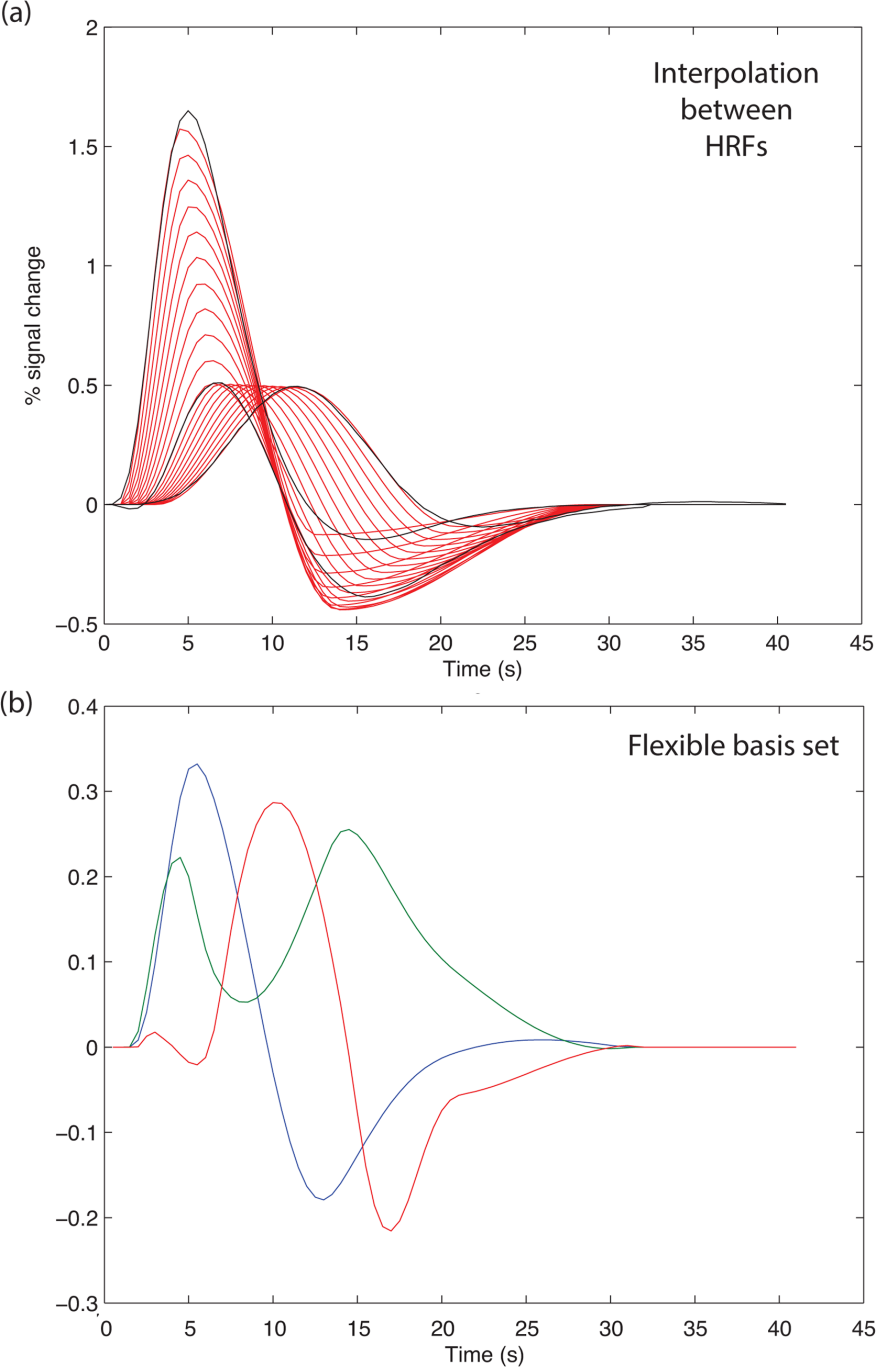
If the HRF for a participant is unknown, a flexible basis set might be useful. (a) We followed the “FLOBs” procedure (see text). The HRFs were parameterized, to allow interpolation of shape between 32 and 38 weeks GA, and between 38 weeks and adults. The black curves show the original HRFs, and the red curves some illustrative interpolated values. (b) Principal components analysis was then used to find a basis set that captured the variance in the interpolated set. Three components captured 99.7% of the variance.

The goal of the flexible basis set is to capture brain activity when the HRF is at some unknown point in the continuum from 32 to 38 weeks GA, or 38 weeks to adult. To capture this range, the HRFs were parameterized and then the parameters interpolated. The resulting set of interpolated HRFs is shown in Fig 4A. The variance in this set was then summarized in a small number of components using principal components analysis (PCA). Three components were found to capture 99.7% of the variance in the interpolated set (Fig 4B).

This flexible HRF basis set recovered signal for all three true HRFs, but gave less power relative to the case where the true HRF is known (see Fig 3, for adults, 32 weeks, 38 weeks mean SPM-T with known HRF 9.41, 4.12, 5.08; with flexible HRF 6.55, 2.85, 3.52). Thus, where the correct HRF is known, using it is preferred, but where there is uncertainty this flexible model is effective.

**Fig 5:**
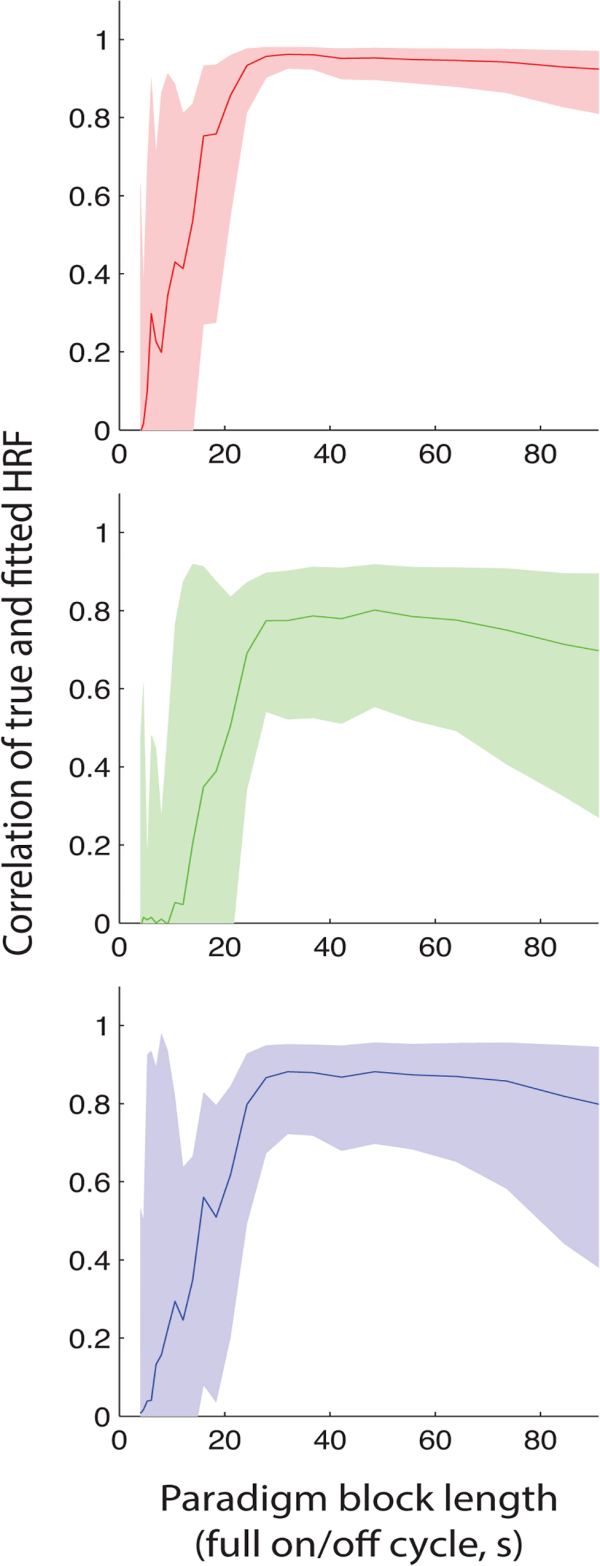
The flexible basis set can be used to estimate the form of the HRF. Here we show the accuracy of the estimation of the HRF for adult, 32 week GA and 38 week GA, as a function of the block design timing (stimulation/rest cycle, x-axis). Correlation was used to assess the similarity of the true (simulated) HRF to the HRF estimated from the flexible model. The mean +/- one standard deviation is shown (calculated in Fisher-transformed space, and then back-transformed).

A further advantage of the flexible basis set is that it allows the HRF to be estimated. Fig 5 shows the quality of this estimation, as a function of stimulation paradigm. For stimulus paradigms with a full on/off periods less than 24s, the HRF was fairly poorly estimated, as the HRF of each block overlaps with the next. For paradigms greater than 60s in period, there are so few onsets that it is difficult to estimate the HRF. In between, the HRF is quite well estimated (adult r>0.93; 32 weeks r>0.69; 38 weeks r>0.80).

For the flexible HRF model, three columns were used. This is displayed as the square root of an adjusted F statistic, which was designed to be comparable to the T statistics, so that similar values correspond to the same *p* values. This was a good approximation everywhere except when there was a very low signal level, as the F statistic is always positive and so unlike the T, it does not have an expected value of zero when averaged across many simulations. To gauge this, we also show in grey the T and F values in a null (no signal) design.

## Discussion

Choosing the correct HRF for modeling was found to be critical for neonatal fMRI. A mismatching HRF can eliminate all power in a block design: in some cases, a mismatched HRF even artifactually yielded a negative signal, but only for short on/off paradigm durations of around 16s. There are a number of reports in the literature of negative fMRI signal in newborns or very young infants [4,6,30–32]. These studies used on/off protocols with cycles greater than 60 s, and so the types of HRF mismatches tested in our modeling cannot explain the negative signal observed. Furthermore, other authors that have shown only positive fMRI signal [5], and so the conditions modulating signal sign are still unclear.

The HRF differentially affected the power of stimulation protocols of different durations. At all three ages, the optimal on/off stimulation period is around 25s. This is in a similar range to the lower end of recommendations for optimal power in adults, of a block length of around 24-60s (Skudlarski et al. 1999; Carter et al. 2008; Dale 1999; http://www.mccauslandcenter.sc.edu/CRNL/tools/fmrisim). However, our modeling shows that for neonates, longer cycles often used in adults yield much less power when the HRF is biphasic at 38 weeks (bottom row, third column of Fig 3). These long designs are affected even more dramatically by a mismatch in the HRF (top row, third column of Fig 3). A key recommendation from this work, therefore, is that slow block designs should be avoided in infants. We suggest 24–30 s for the full on/off cycle.

Given the strength of the effect of the HRF on the power of fMRI designs, and the extent to which it changes through the age range, it is important for future work to replicate, generalize and extend Arcihi et al’s (2012) measurements of the HRF to a somatosensory stimulus. It will be useful to characterize: the robustness of these results; degree of variability across individuals in the HRF as a function of age (i.e., cross-sectionally); the effect of brain injury on the development of the HRF; and whether the HRF develops at the same rate in all brain regions.

The flexible basis set was effective in recovering signal from the diverse range of HRFs tested. The PCA analysis showed it captured almost all of the variance from Arichi’s three HRFs, and the temporally-interpolated HRF set, using a linear model with just three components. This simple model will be more robust than a more general (e.g., finite impulse response, Lee et al, 2012) basis sets that has a greater number of degrees of freedom. However, they will fail if an infant has an HRF distinct from Arichi’s set.

In sum, using simulations with an accurate noise model we found that stimulation paradigms for newborn fMRI must be carefully chosen, as the power is optimal for a narrower range of paradigms than for adults. For block designs, we recommend full on/off cycle durations of 20-30s. Furthermore, we have shown that it is important to analyze newborn data with the correct HRF, or to use a flexible basis set where the correct HRF is unknown.
